# Flexible Acceptance Condition of Generics from a Probabilistic Viewpoint: Towards Formalization of the Semantics of Generics

**DOI:** 10.1007/s10936-022-09851-1

**Published:** 2022-08-21

**Authors:** Soo Hyun Ryu, Wonsuk Yang, Jong C. Park

**Affiliations:** 1 School of Computing, Korea Advanced Institute of Science and Technology, Daejeon, Korea; 2 Present Address: Department of Psychology, University of Michigan, Ann Arbor, MI USA

**Keywords:** Generics, Generalization, Acceptance condition, Formal semantics

## Abstract

Formalization of the semantics of generics has been considered extremely challenging for their inherent vagueness and context-dependence that hinder a single fixed truth condition. The present study suggests a way to formalize the semantics of generics by constructing flexible acceptance conditions with comparative probabilities. Findings from our in-depth psycholinguistic experiment show that two comparative probabilities—cue validity and prevalence—indeed construct the flexible acceptance conditions for generics in a systematic manner that can be applied to a diverse types of generics: Acceptability of *IS_A* relational generics is mostly determined by prevalence without interaction with cue validity; feature-describing generics are endorsed acceptable with high cue validity, albeit mediated by prevalence; and acceptability of feature-describing generics with low cue validity is mostly determined by prevalence irrespective of cue validity. Such systematic patterns indicate a great potential for the formalization of the semantics of generics.

## Introduction

Generics are the sentences expressed to deliver generalized information about a particular category, such as “*Cats like milk*.”^1^ Such statements play a vital role in our daily lives, since they contribute to facilitating children’s concept development (Cimpian & Markman, 2009; Gelman, 2009; Leslie, 2007, 2008) and conveying prototypical and/or essential properties of categories (Declerck, 1986; Geurts, 1985; Heyer, 1985; Nunberg & Pan, 1975; Platteau, 1980). Despite the importance and frequent usage of generics, however, little has been agreed upon their meaning, failing to obtain the formalized semantics which is understood through the lens of truth condition (Davidson, 1967; Montague, 1973; Tarski, 1956). In this paper, we conjecture that establishing truth-conditional semantics of generics has been unavailable due to their two properties: *vagueness* and *context-sensitivity* (Nguyen, 2020; Sterken, 2015a; Tessler & Goodman, 2019; van Rooij & Schulz, 2020). To be specific, we see that generics fall into a type of sentences that have vague boundaries to receive the dichotomous truth values (true or false), which therefore prompt the necessity of considering graded truth values referred to as ‘acceptabilities.’

In addition, we also note that even when the gradability is taken into account, formalizing the semantics of generics still remains puzzling, because the condition that determines acceptability is highly context-sensitive. In other words, there seems no single condition that guarantees either an increase or a decrease in the acceptability of all kinds of generics. The details of generics’ properties that hinder the formalization of their semantics will be laid out in the following section.

Given the inherent properties of generics that preclude the characterization of their meaning with a single fixed truth condition, the present study aims to suggest an alternative way to formalize their semantics by exploiting the flexible acceptance condition defined in terms of comparative probabilities (Cohen, 1996, 1999). To this end, we conduct a psycholinguistic experiment to closely investigate the way the acceptability of generics is determined with respect to comparative probabilities varying to the context. The results of the experiment provide evidence that the acceptability of generics can be specified in a flexible but systematic way, shedding light on the possibility of formalization of the semantics of generics in terms of acceptance condition.

We acknowledge that semantics and acceptability are arguably distinct and that, therefore, separate analyses should be conducted for each of them. In particular, the error theory of generics (Sterken, 2015a, b) points out that acceptance of generics is sometimes endorsed only due to a cognitive bias (e.g., sensitivity to strikingness), regardless of their truth-conditional semantics. Thus, it assumes that the determination of the generics’ acceptability should be understood as byproducts of cognitive process in sentence comprehension, which stand separate from the semantics. Although this account is intriguing and deserves further investigation, we postulate that, at least when it comes to generics, the semantics definitely needs to be examined in association with acceptability. This is mainly because we base our analysis of generics on the belief that the primary function of generics is to make a generalization of a category, which obviously involves a lot of cognitive processes (Leslie, 2008; Leslie & Lerner, 2016). If the meaning mainly delivered by generics reflects our cognitive process, then what can we learn about its semantics without taking the cognitive process into account? In this regard, this study maintains that the formalization of the semantics of generics needs to be studied in terms of acceptability. At the same time, however, we also believe that further investigation into how the acceptability and the semantics may stand together will certainly deepen our understanding of generics.

The remainder of the paper is structured as follows. In “Why Is It So Challenging to Formalize the Truth-Condition of Generics? Motivation for Acceptability-Conditional Semantics” section, we discuss why it is difficult to formalize the semantics of generics in terms of truth condition, focusing on generics’ vagueness and context-sensitivity. In “Previous Studies” section, we review a diverse range of previous theories that have attempted to account for the condition that renders generics acceptable together with their respective weaknesses, and then examine why the probabilistic approach has a better potential to characterize the semantics of generics. In “The Present Study” section, we introduce our psycholinguistic experiment where the acceptability of generics and two comparative probabilities are measured. “Results and Discussion” section shows the results and discussion where the relations among metrics are investigated in order to construct the flexible acceptance conditions for generics. In “General Discussion” section, based on the empirical data obtained from the experiment, we summarize our findings of how the two comparative probabilities interact to determine the acceptability of generics in different context, claiming that the meaning of generics can also be understood through the formalized semantics with the flexible acceptance condition.

## Why Is It So Challenging to Formalize the Truth-Condition of Generics? Motivation for Acceptability-Conditional Semantics

As briefly stated in the previous section, the semantics of generics is hard to formalize with a single fixed truth condition due to their vagueness and context-sensitivity (Sterken, 2015a; Tessler & Goodman, 2019; van Rooij & Schulz, 2020). In this section, we look more closely into how such properties preclude truth-conditional semantics of generics, motivating our present approach to the semantics of generics: flexible acceptance condition of generics.

### Vagueness of Generics

A truth condition for a given sentence specifies under which situation it is determined to be true. Knowing the truth condition has been considered an integral part of understanding the meaning of the sentence, at least from the perspective of formal semantics (Davidson, 1967; Tarski, 1956). However, such logical tools are not applicable to all the sentences that natural language can generate. Rather, understanding the meaning of a sentence through the truth condition is merely limited to a partial portion of sentences generated in our daily lives since fuzziness and vagueness are pervasive in natural language (Hong, 2021).

The *Sorites paradox* (i.e., the paradox of the heaps) well describes how such vagueness of sentences can put truth-conditional semantics in trouble, which represents situations where there are no clear-cut conditions to determine whether a sentence is completely true or completely false. The paradox illustrates that even three grains can be considered as a heap when using Modus Ponens in (1): Starting from the base premise (1a), multiple applications of the inductive premise (1b) can eventually lead to the infelicitous conclusion (1c)

1. *Sorites Paradox*
  - *base premise*
    A million of grains make a heap.
  - *inductive premise*
    If *N* grains make a heap, then *N – 1* grains also make a heap.
  - *conclusion*
    Three grains make a heap.

Even though the inductive premise (1b) sounds reasonable enough since a single grain does not contribute much to forming a heap, the repeated application of Modus Ponens between 1a and 1b should surely stop at a certain point in order to maintain the truth of the conclusion. Without such a limit at a proper point, it ends up with absurdity such as (1c).

In a similar logic, generics encounter the same type of paradox as shown in the argument in (2) where the false conclusion (2c) can be reasoned from the base premise (2a) by repeatedly applying Modus Ponens to the inductive premise (2b).

(2) *The Paradox of generics*
  - *base premise*
    The fact that 100% of cats like milk renders a statement “*Cats like milk*” true.
  - *inductive premise*
    If the fact that *N*% of cats like milk renders a statement “*Cats like milk*” true, then the fact that *N-1*% of cats like milk also renders a statement “Cats like milk” true.
  - *conclusion*
    The fact that *1*% of cats like milk also renders a statement “*Cats like milk*” true.

As shown in the paradox above, there is no certain boundary that specifies from which point an application of the inductive premise leads to losing the truth of the conclusion. Rather, it seems that the degree of the truth of such sentences gradually decreases as the inductive premise is continuously applied, the sentences being partially true and partially false at the same time.

In this regard, the semantics of generics is not reducible to a single truth condition since the truth value of the conclusion in the paradox (2) should be viewed with ‘gradability’: The degree of truth of the conclusions can gradually change. Given such limitation of truth-conditional semantics in explaining generics, we exploit ‘acceptance conditions’ that integrate the vagueness of such sentences with logical perspective of truth-conditional semantics. In such an approach, instead of the dichotomous truth-conditional values that are represented as either *0* or *1*, the acceptability of the sentences is judged on a gradable scale enabling the expression of “how much acceptable a sentence is as true.” in a continuous manner^2^.

### Context-Sensitivity of Generics

The vagueness of generics is not the only aspect that hinders the formalization of the semantics of generics, due to the context-sensitivity of the condition that determines the acceptability of generics (Nickel, 2008; Sterken, 2015a). Let’s examine the Sorites paradox again. The likelihood that a group of sand is considered as a heap linearly increases as the amount of sand goes up. However, such linearity is not invariably observed across all types of generics. If more inductive premise in the argument (2) being applied necessarily leads to the lower acceptability of generics regardless of the context, then it would predict higher acceptability in (3a) than in (3b) since what each sentence actually implies, based on our basic world knowledge, are (3c) and (3d) that require approximately 10 and 50 applications of the inductive premise (such as (2b)), respectively.

(3)
  - ?People are right-handed.
  - Ducks lay eggs.
  - (*Approximately 90% of people are right-handed, and we say that ...*)
    ? People are right-handed.
  - (*Approximately 50% of ducks lay eggs, and we say that ...*)
    Ducks lay eggs.

In contrast, however, people tend to find (3b) more acceptable than (3a), although it is assumed that more inductive premises are applied for (3b) exemplifying how inconsistent the acceptance condition for generics can be.

The fact that generics do not specifically show consistent similarity to any of the sentences with a generalized quantifier, such as *all*, *most*, or *some* (Lewis, 1975) further illustrates the context-sensitivity of the acceptance condition of generics. If the number of times the inductive premise is applied before getting to the conclusion is what affects the acceptability of generics, then the similarity of generics to *some-*quantified sentences, *most-*quantified sentences, and *all-*quantified sentences should increase linearly since the proportion of the entities that each quantifier specifies increases and thus necessitates a fewer number of inductive premises in such order (Barwise & Cooper, 1981). However, the following examples describing how the sentences with each quantifier can be clearly distinct from generics show that the acceptability of generics cannot be explained with a single fixed condition.

First, the fact that people easily accept “*Some Canadians are left-handed*”, while rejecting “*Canadians are left-handed*”, illustrates some discrepancy of semantics between *some*-quantified sentences and generics. Likewise, the acceptability of “*Ducks lay eggs*”, contrary to the infelicitous nature of “*Most ducks lay eggs*”, evinces that *most*-quantified sentences are distinct from generics. In addition, the sentence “*All tigers are striped*” does not hold because of some unusual tigers with no stripes, while the sentence “*Tigers are striped*” sounds felicitous enough. Therefore, *all*-quantified sentences cannot also be considered similar to generics. Provided that any of quantified sentences do not show invariable commonality with generics, we confirm that the acceptability of generics is flexibly determined unlike the way quantified sentences are explained in terms of fixed logical set inclusion relations (Barwise & Cooper, 1981; Schubert & Pelletier, 1987).

To summarize, these examples well describe how mysterious and context-sensitive the semantics of generics is since there is not a single condition that can be applied to account for the acceptability of all kinds of generics.

## Previous Studies

Drawn by such uniqueness and complexity of generics , scholars from many disciplines—linguistics, philosophy, psychology and cognitive science—have attempted to provide a solid explanation that accounts for the conditions that license all types of generics. In this section, we provide a brief coverage of representative previous attempts. For more detailed discussion about the previous studies, we refer readers to Leslie and Lerner (2016).

### Diverse Approaches to the Semantics of Generics

First, normalcy-based approaches (Nickel, 2008) maintain that the acceptability of generics is determined based on the assumption of the situation being as normal as possible. According to this point of view, the reason we accept a sentence ‘*Tables are flat*’ can be explained with the fact we may expect a table to be flat in the normal world where we are living. However, numerous counterexamples show the limitation of such approaches: In normal cases, most ticks do not carry Lyme disease, though people easily accept the generics ‘*Ticks carry Lyme disease*.’

Situation semantics-based approaches (Barwise & Perry, 1981; Cavedon & Glasbey, 1994; Ter Meulen, 1986) assume that only relevant entities are taken into account to estimate the acceptability of generics. For example, when determining the acceptability of the sentence ‘*Ducks lay eggs*,’ only the ones that have the ability to give birth are taken into account since they are the entities especially relevant to the interpretation of the sentence. Although enabling to consider the context-sensitivity while interpreting the semantics of generics, such approaches seem to be still in their infancy since the logic to specify relevant entities has not yet been fully developed.

Stereotypical and prototypical approaches are another way to explain the meaning of generics (Declerck, 1986; Geurts, 1985; Heyer, 1985; Nunberg & Pan, 1975; Platteau, 1980; van Rooij and Schulz, 2020). According to these approaches, the acceptability of generics is determined based on the degree to which the delivered message is compatible with comprehenders’ stereotype and prototype of the entity being discussed in the generics. For instance, the reason why people easily accept the generics ‘*Lions have manes*’, despite the fact that less than half of the lions are with manes, is that the prototype of the lion in people’s mind is the one with manes. However, this does not provide exhaustive accounts since not all generics behave in association with typicality. For instance, people are likely to reject a sentence ‘*Books are paperbacks*’, even though a member of a category ‘*book*’ is typically a ‘*paperback*’, a case that cannot be explained with typicality-based approaches.

Many previous attempts, including aforementioned approaches, have deepened our understanding of generics having respective strengths and weaknesses. Despite such diversity in previous efforts to explain the semantics of generics, one critical shortcoming is commonly found, at least from the perspective of the formal semantics: There is no objective tool to set criteria for the acceptability estimation that can be uniformly applied to a diverse types of generics, which would be indispensable to formal semantics.

Unlike other approaches, however, probabilistic approaches suggest a way to understand the meaning of a broad range of generics in a systematic and objective manner. Specifically, they claim that comparative probabilities—*prevalence* and *cue validity*—can systematically account for the acceptability of generics (further explanation of which will be provided shortly), and therefore the semantics of generics may be formalized in terms of probabilistic values (Cohen, 1996, 1999, 2004, 2009; Khemlani et al., 2012; Leslie, 2007; Prasada et al., 2013; van Rooij & Schulz, 2020; Tessler & Goodman, 2019). In this regard, we believe that the probabilistic approach is of special importance for its promise to establish a formalized account of the flexible semantics of generics among various approaches that attempted to explain the semantics of generics.

### Probabilistic approaches to the semantics of generics

Attempts to explain the semantics of generics in terms of probabilities date back to Cohen’s studies 1996, 1999, 2004, 2009. Given the complex nature of the generics, Cohen (2009) acknowledged that generics are one kind of sentences that cannot be fully explained with a truth-conditional account. Rather, unlike some sentences that can be judged with a single and rigid truth condition, he noticed, generics are a type of sentences that heavily rely on gradability to judge their truthiness which can be represented in terms of probabilities, and he suggested two types of generics that exploit different kinds of probabilities: absolute generics and relative generics.

Absolute generics are those that hold true under the following condition:

(4) “*Ks are F*” is true iff the probability that an arbitrary *K* that satisfies some predicate in Alt(*F*) satisfies “is *F*” is greater than .5.

Specifically, this condition renders generics (“*Ks are F*”) acceptable when the *prevalence*—the probability of the particular category (*K*) having the feature (*F*)—chosen among alternative features (Alt(*F*)) that are relevant to the context—is greater than 0.5. For instance, suppose “*Tigers are striped*.” Among the relevant predicates in Alt(*F*) that describe the pattern of animal fur, such as “*being plain*”, “*being spotted*”, or “*being striped*”, that the probability of a tiger being striped is greater than 0.5 renders the sentence “*Tigers are striped*” acceptable.

However, such approach is incomplete since there remain myriads of generics that cannot be felicitously expressed, even satisfying the condition of absolute generics, as listed in (5).

(5)
  - ?Bees are workerbees.
  - ?Books are paperbacks.

In addition, there are also cases where the generics are felicitously accepted while failing to satisfy the condition of absolute generics, as listed in (6).

(6)
  - Mosquitoes carry West Nile virus.
  - Sharks attack swimmers.

The generics whose acceptability cannot be explained in terms of absolute probability are named relative generics according to Cohen (1996). The acceptability of relative generics is explained in terms of *cue validity*—the conditional probability that an object falls in a particular category given a particular feature or cue (Beach, 1964; Cohen, 1996). Specifically, the condition that renders relative generics acceptable is clarified in (7).

(7) “*Ks are F*” is true iff the probability that an arbitrary *K* that satisfies some predicate in Alt(*F*) satisfies “is *F*” is greater than the probability that an arbitrary member of Alt(*K*) that satisfies some predicate in Alt(*F*) satisfies “is *F*”.

Under this condition, the acceptability of the sentence (6a) can be easily accounted for despite its low prevalence: Mosquitoes are more likely to carry West Nile virus than the other relevant categories in Alt(*K*).

To date, a number of follow-up studies have shown that the interplay between prevalence and cue validity plays a critical role in determining the acceptability of generics (Khemlani et al., 2012; Kochari et al., 2020; Leslie, 2007; Prasada et al., 2013). Particularly, recent studies (Tessler & Goodman, 2019; van Rooij & Schulz, 2020), relevant to Cohen’s relative accounts of generics, proposed powerful models capable of predicting human acceptability ratings on generics by measuring how *typical* or *representative* a certain feature is for a category. Despite their strong predictive power and psychological plausibility, however, these probabilistic models still seem to be quite restricted to certain types of generics that describe typicalities and stereotypes, not being able to cover a broad range of types of generics.

Although previous probabilistic approaches have opened the door to the formalization of the uniform semantics of generics, attempting to find consistent conditions that render generics acceptable, they still show limitations in formalizing the semantics of generics, missing a detailed and systematic explanation of how the acceptability and comparative probabilities are exactly related to each other, and under what circumstances each probabilistic metric plays a major role in determining the acceptability of generics.

In order to overcome the limitations and to deepen our understanding of the semantics of generics from the probabilistic perspective, we conduct an experiment whose main purpose is to examine the influence of prevalence and cue validity on the acceptability of generics in diverse contexts and eventually to construct flexible acceptance conditions of generics. We believe that the present study advances probabilistic accounts of generics for two reasons. First, the explicit relation between prevalence and cue-validity is examined to give a clearer understanding of the condition where generics are accepted. Second, we look deeper into the acceptance conditions that can be applied to a broad range of types of generics.

## The Present Study

We explored the flexibility of the semantics of generics, which was especially made explicit while attempting to explain the acceptance condition of generics in probabilistic terms. In particular, it was noted that some cases rely on prevalence in determining the acceptability of generics while other cases rely on cue validity. Despite such flexibility of the acceptance condition, however, we believe that there exist systematic patterns of the way in which comparative probabilities determine the acceptability of generics and that knowing such patterns will eventually lead us to a deeper understanding of the semantics of generics, which may result in its formalization. Along this line, we carried out an experiment where the conditions in which prevalence and cue validity influence the acceptability of generics were explored in great detail. To be specific, we measured the acceptability of generics and the comparative probabilities relevant to each generics—prevalence and cue validity—that language users exploit to estimate the acceptability of generics, and then investigated under what contexts two probabilities put how much influence on the acceptability of generics. The results of the experiment showed that the distinct influence of prevalence and cue validity on the acceptability of generics hinges on contexts in a systematic way.

The experiment was designed primarily following the previous studies (Khemlani et al., 2012; Prasada et al., 2013; Tessler & Goodman, 2019), with a few minor but critical modifications: The present study included an additional step to determine the superordinate category of the target category of each generics and the present study induced participants to rate prevalence using their expectation-based reasoning rather than world knowledge.

### Ratings

#### Acceptability

The acceptability of each generics was rated on a 7-point Likert scale, − 3 being “Definitely reject”, 0 being “Not sure” and +3 being “Definitely accept.”

#### Expectation-Based Prevalence

The questions for prevalence measurement are to obtain individuals’ prior probabilistic knowledge of $P({x} \in {F} | {x} \in {K})$, which is the probability of *x* having feature *F* given that it is a member of category *K*. Though prevalence has been also asked in previous studies (Khemlani et al., 2012; Prasada et al., 2013; Tessler & Goodman, 2019), the design of the present study for prevalence estimation was quite different from those of the previous studies in that we did not lead participants to rely on the exact world knowledge for the prevalence estimation as previous studies. Instead, we induced participants to rate the prevalence based on their expectation of some category, which probably has been constructed through their personal experience. To be specific, for the generics “*Ducks lay eggs,*” the question in the previous studies for prevalence estimation, “*What percentage of ducks lay eggs*?”, was replaced with “*Suppose that you encounter a duck X. How likely is it that X lays eggs*?” in our study.

Such modification is motivated by the fact that what people *know* about the world is not always compatible with what they actually *experience* in their daily lives. For example, suppose that “*Lions have manes*.” In the real world, much fewer than 50% of lions have manes since only mature and male lions are with manes. However, such world knowledge is misaligned with people’s empirical knowledge that has been accumulated based on personal experiences because a significant proportion of images of lions in daily lives are the ones with manes. Any remaining doubt about it can be resolved by looking up the images of lions on the Internet, which will clearly show how unsuccessful our personal experience may be in reflecting the actual world knowledge. Along with the belief that an individual world experience highly impinges on linguistic knowledge (Bergen, 2015), we assumed that such individual expectation-based reasoning may outweigh individuals’ world knowledge in determining the acceptability of generics.

The expectation-based prevalence was rated on a 0–100 scale. Henceforth, we will refer to “expectation-based prevalence” as “prevalence” for the sake of brevity.

#### Superordinate Category that Encompasses the Alternative Categories

Cue validity, which is one type of comparative probabilities that influences the acceptability of generics, refers to the conditional probability of an entity falling into a specific category given that it has a particular feature. In this regard, comparison with alternative categories that can have the same particular feature is critical to the determination of cue validity, and therefore knowing what alternative categories are taken into account for the evaluation of the acceptability of generics is indispensable for the reliable rating of cue validity.

An example generics “*Dolls wear dresses*” reveals the importance of considering alternative categories with respect to the given feature while estimating cue validity. If the alternative categories are not specified at all, then the cue validity would be rated low since the feature “*to wear dresses*” can belong to many other categories, such as princesses or school girls, rather than to dolls alone. In contrast, if alternative categories considered for the feature “*to wear dresses*” are other kinds of toys, then the cue validity for the given generics may be rated high since the likelihood of dolls wearing dresses is high when compared with other kinds of toys, such as balls, robots or cars.

Despite the importance of knowing members of which superordinate category are considered as the alternative categories for specific generics, it is hard to know what alternative categories are considered for each generics because alternative categories are selected depending on sentential contexts (Schubert & Pelletier, 1989). Moreover, it is also implausible to simply select the hypernym of the target category as the superordinate category of alternative categories for the generics given the fact that people construct a category taxonomy based on the way they interact with the world, regardless of how categories are actually constructed in the world (Rosch et al., 1976).

Thus, it is essential to find out the superordinate category that encompasses the alternative categories considered for each generics and provide it as a reference point for cue validity rating, even though previous studies lacked this process. Therefore, we asked participants to answer members of which superordinate category are considered for the acceptability estimation of each generics, so that we can provide a fair and reliable reference point for the process of cue validity rating. We considered the most frequently mentioned superordinate category as the reference point for cue validity rating task for each generics.

#### Cue Validity

The questions for cue validity measured prior probabilistic knowledge of $P({x} \in {K} | {x} \in {F})$, which qualifies the validity of *x* being a member of category *K*, given that it has feature *F*.

In the questions for cue validity rating, *x* was rendered to be a member of superordinate category *K*, which is determined according to the description in the previous section. For instance, the majority of people responded that they intuitively took into account members of category *toy* for the generics “*Dolls wear dresses*.” Correspondingly, for this target generics, cue validity was measured with the question “*Suppose that* ***a toy X*** *wears dresses. How likely is it that* ***X*** *is a doll?”*, instead of “*Suppose that ****X*** *wears dresses. How likely is it that* ***X*** *is a doll?”* that is used in previous studies. The results of the experiment corroborated that the additional process of determining the superordinate category of a target category significantly affects cue validity ratings. Specifically, the cue validity for ‘*Dolls wear clothes*’ in the present study was rated as 2.00, much higher than 0.47, which is the rating from previous study (Prasada et al., 2013).

The cue validity was rated on a 7-point Likert scale, − 3 being “Very unlikely”, 0 being “Not sure”, and +3 being “Very likely.”

### Method

#### Participants

Using Amazon Mechanical Turk, we recruited 456 participants whose IP addresses were restricted to those of the United States. 360 participants were assigned to the first task where the acceptability and superordinate category of generics were determined. For the first task, the average time spent on the task was 7 minutes and the participants were rewarded with $1.

A group of 96 participants were assigned to the second task where prevalence and cue validity were measured. The average time spent was 12 minutes and the monetary reward for the participants was $2.

#### Materials

The 84 target generics from Prasada et al. (2013) were used for the diversity of generics types they cover. Among the target generics, 60 statements are generally considered acceptable, while 24 statements are considered unacceptable. For each generics, four measurements—acceptability, prevalence, superordinate category, and cue validity—were employed throughout two tasks of the experiment.

#### Procedure

The experiment consisted of two subtasks, completed by different groups of participants. In the first task, participants were randomly assigned with seven generics accompanied by two questions regarding the acceptability and the superordinate category intuitively considered when estimating the acceptability of generics. Participants were provided with a slider bar to rate the acceptability. The example questions for the first task are provided in Table 1.

**Table 1:** Questions of the first task for the generics “*Ducks lay eggs*”

Type	Question	Scale
		7-point Likert scale
Acceptability	Please rate how acceptable the following sentence is “*Ducks lay eggs*”	(+3 = “Definitely accept”, 0 = “Not sure”, − 3 = “Definitely reject”)
Subordinate category	When you estimate the acceptability of “Ducks lay eggs,” members of what superordinate category were considered for comparison?	
	For example, when you assessed “Bananas are sweet,” you could have compared bananas with apples, oranges, or melons. In this case, your superordinate category is “*fruit*”	
	If your judgment did not rely on comparison, please answer “*N/A*”	

In the second task, the participants were asked to rate either prevalence or cue validity, on randomly assigned 42 generics. Participants were also provided with a slider bar to provide their answers. The example questions for the second task are as shown in Table 2.

#### Ethics

The experiment was approved by Korea Advanced Institute of Science and Technology (KAIST) IRB (KH2018-80), and the informed consent was read and acknowledged by participants prior to their tasks. Since the experiment was run on the AMT platform where workers are required to be at least 18-years-old, obtaining surrogate consents was not applicable to this study.

**Table 2:** Question of the second task for the generics “Ducks lay eggs”

Type	Question	Scale
Prevalence estimation	Suppose you encounter a duck X	0–100
	How likely is it that X lays eggs?	
		7-point Likert scale
Cue validity rating	Suppose a bird X lays eggs	(+3 = “Very likely”,
	How likely is it that X is a duck?	0 = “Not sure”,
		− 3 = “Very unlikely”)

## Results and Discussion

Before analyzing the data, data points that do not fall within two standard deviations of the mean of each rating were considered outliers and thus removed.

We first noticed that the target generics in the experiment are mainly divided into two subtypes that function differently: (1) *IS_A* relational generics that serve to represent the taxonomic relationship between two categories and (2) feature-describing generics that serve to generalize the feature of particular categories.

**Fig. 1:**
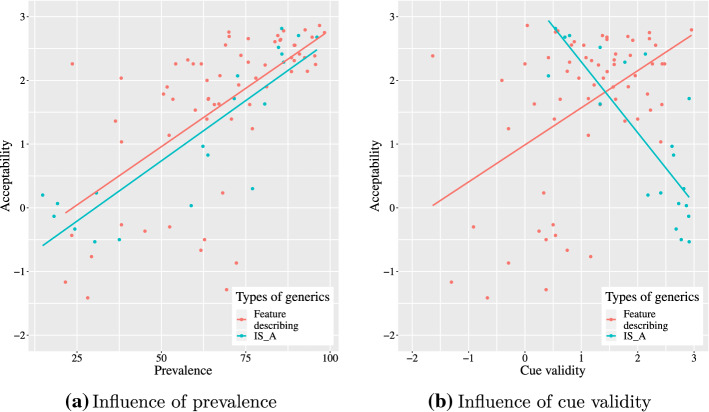
Influence of comparative probabilities on the acceptability of generics by types of generics

The distinctiveness between *IS_A* relational generics and feature-describing generics is confirmed in terms of the relation between the acceptability and comparative probabilities as illustrated in Fig. 1,^3^. According to the figure, (a) shows that the acceptability is positively correlated with prevalence of both *IS_A* relational generics (*r*(18) = .87, *p* < .001) and feature-describing generics (*r*(62) = .62, *p* < .001) while (b) shows that there is an opposite influence of cue validity on the acceptability depending on whether the subtype of generics is *IS_A* relational or feature-describing. In particular, cue validity positively correlates with the acceptability of the feature-describing generics (*r*(62) = .62, *p* < .001), while negatively correlates with the acceptability of *IS_A* relational generics (*r*(18) = − .81, *p* < .001). Given such clear distinctive patterns of the two types of generics, we treated them individually and separate analyses for each subtype of generics were conducted.

### *IS_A* Relational Generics

For the *IS_A* relational generics, high negative correlation between prevalence and cue validity was revealed (*r*(18) = − .64, *p* < .01). Such negative correlation is valid enough, given the property of the *IS_A* relational generics that delivers a taxonomic relation between hyper-category and hypo-category. For the acceptable generics where a hypo-category is claimed to be a sort of hyper-category, e.g., “*Dogs are mammals*,” the prevalence estimation, which is rated with a question “*Suppose that you encounter a dog X. How likely is it that X is a mammal?*” should be rated high since all dogs taxonomically belong to mammals. Likewise, the cue validity measured with a question “*Suppose that an animal X is a mammal. How likely is it that X is a dog?*” is to be rated low, given that there are many other kinds of animals classified as mammals other than dogs. In a similar line, for the inverse version of generics, e.g., “*Mammals are dogs,*” the two metrics are rated in the opposite way, prevalence being rated low and cue validity being rated high.

Provided the natural existence of a close negative correlation between prevalence and cue validity, which is attributed to the inherent property of *IS_A* relational generics, the acceptability of *IS_A* relational generics can be simply explained with either prevalence or cue validity, without any necessity to presuppose an interactive relation between the two probabilities. Indeed, both probabilistic metrics show a high correlation with the acceptability, even though the explanatory power of prevalence (*r*(18) = .87, *p* < .001) was slightly greater than the one of cue validity (*r*(18) = -.81, *p* < .001). Therefore, we conclude that the acceptability of *IS_A* relational generics can be explained with prevalence, without any interaction with cue valence. The list of the *IS_A* relational generics and their metrics is provided in Table 3.

**Table 3:** The metrics for the generics that are IS_A relational

Target generics	Acceptability	Prevalence	Cue validity
Dogs are mammals	2.81	85.65	.54
Ants are insects	2.70	90.63	.79
Cats are animals	2.68	96.10	.70
Rectangles are geometric figures	2.52	84.70	1.33
Kangaroos are marsupials	2.41	85.71	2.14
Mushrooms are fungi	2.29	86.23	1.77
Elms are trees	2.07	72.58	.41
Computers are PCs	1.71	71.58	2.91
Sows are pigs	1.63	80.62	1.33
Books are paperbacks	.97	62.30	2.61
Mammals are placental mammals	.83	63.78	2.64
Bees are workerbees	.30	77.00	2.82
Houses are mansions	.23	30.74	2.41
Mammals are hamsters	.20	14.82	2.18
Dogs are beagles	.06	19.22	2.73
Trees are deciduous trees	.03	58.83	2.86
Trees are palm trees	− .13	18.18	2.90
Plants are ferns	− .33	24.35	2.68
Novels are mysterious novels	− .50	37.54	2.77
Restaurants are Chinese restaurants	− .53	30.26	2.91

### Feature-Describing Generics

No significant correlation between prevalence and cue validity was found for feature-describing generics (*r*(62) = .11, *p* $=$ .37), contrary to *IS_A* relational generics. According to the results of the multiple linear regression, the main effects of both prevalence (*t* $=$ 6.937, *p* < .001) and cue validity (*t* $=$ 4.02, *p* < .001) and interaction between the two predictors (*t* $= -2.44$, *p* < .05) were found as summarized in Table 4. In order to account for the interaction effect, we divided the feature-describing generics into two groups by the value of cue validity.

**Table 4:** Summary of multiple regression analysis for predicting the acceptability of generics

	Estimate	SE	*t* value	Pr ($>|t|$)
(Intercept)	− 1.81	.43	− 4.25	<.0001
Prevalence	.04	.01	6.94	<.0001
Cue validity	1.16	.29	4.02	<.0001
Prevalence $\times$ Cue validity	− .01	.00	− 2.44	<.05

#### Feature-Describing Generics Whose Cue Validity Is High

30 statements with cue validity higher than 1.3 were selected as generics with high cue validity. As shown in Table 5, all the generics with high cue validity are accepted, even when the prevalence value is as low as 23.

**Fig. 2:**
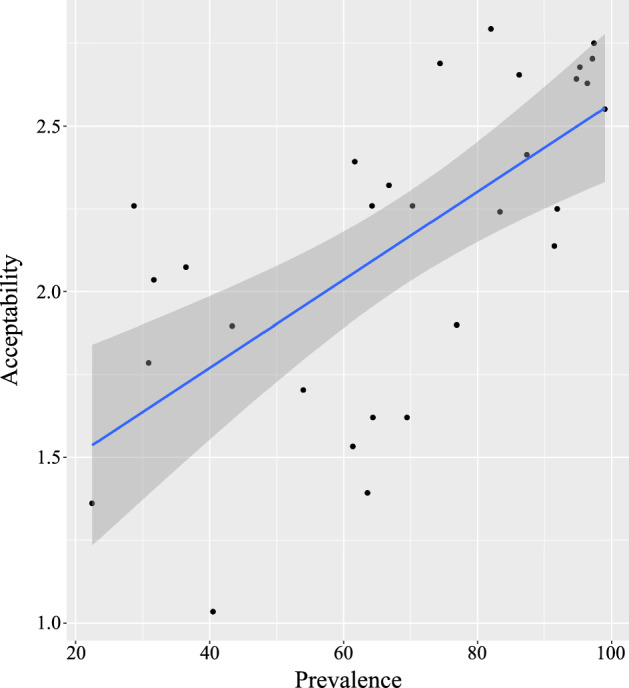
Influence of prevalence on the degree of the acceptability for high cue validity generics

Even though high cue validity endorses generics acceptable, this, however, does not necessarily mean that prevalence puts no impact on the determination of the acceptability. Rather, we find that prevalence shows a significant positive correlation with the acceptability for the generics with high cue validity (*r*(28) = .62, *p* < .001) (see Fig. 2) while cue validity does not show any further relation with the degree of the acceptability (*r*(28) = − .03, *p* $=$ .86). In summary, even though high cue validity principally guarantees generics to be accepted, the degree of the acceptability of the generics with high cue validity is marginally predicted by prevalence.

**Table 5:** The metrics for the generics whose cue validity is high

Target generics	Acceptability	Prevalence	Cue validity
Even numbers are divisible by 2	2.79	92.56	2.95
Birds have wings	2.75	98.33	2.21
Ambulances have sirens	2.70	84.50	1.35
Cows have udders	2.69	70.10	1.83
Leopards have spots	2.68	94.67	1.45
Kangaroos have pouches	2.66	75.74	2.21
Scissors cut	2.64	85.35	1.45
Bachelors are unmarried	2.63	85.17	1.91
Airplanes have wings	2.55	89.36	2.32
Cars have radios	2.41	90.91	1.61
Knives cut people	2.39	73.57	1.87
Deer have antlers	2.32	57.83	1.58
Dogs bark at strangers	2.26	61.83	2.48
Mosquitoes carry Malaria	2.26	23.61	2.26
Winters are snowy	2.26	59.43	2.43
Lemons are sour	2.25	95.68	1.58
Cats like milk	2.24	80.74	1.30
Tables are flat	2.14	88.55	1.46
Snowstorms shut down schools	2.07	67.35	1.96
Car crashes kill people	2.04	38.09	1.39
Peacocks have beautiful tails	1.90	81.17	1.83
Guns kill people	1.90	51.71	1.57
Ticks carry Lyme disease	1.78	50.62	2.17
Rusty nails cause tetanus	1.70	53.42	1.61
Raccoons eat garbage	1.62	76.17	1.33
Summers are humid	1.62	65.70	2.43
Lions have manes	1.53	60.04	2.23
Dolls wear dresses	1.39	63.48	2.00
Rip tides drown swimmers	1.36	36.39	1.77
Loud noises deafen people	1.03	38.17	2.41

#### Feature-Describing Generics Whose Cue Validity Is Not High

For the generics which have less than 1.3 of cue validity (see Table 6), we ran two correlation analyses, one for the relation between acceptability and prevalence and the other for the relation between acceptability and cue validity. The results of correlation analyses showed that the acceptability of generics whose cue validity is not high is mostly determined by prevalence (*r*(32) = .74, *p* < .001), having no more influence from cue validity (*r*(32) = .31, *p* = .08).

Despite the strong correlation between the acceptability and prevalence, several counterexamples, whose acceptability is barely explained in terms of prevalence, still remain unanswered. Most notably, some generics show lower than zero acceptability even with its high prevalence, which is greater than 60, as listed in (8).

(5)
  - ?Teachers are female.       (Acceptability = − .50, Prevalence = 62.79)
  - ?Humans are over 3 years old.       (Acceptability = − .67, Prevalence = 61.71)
  - ?Canadians are right-handed.       (Acceptability = − .87, Prevalence = 72.22)
  - ?Engineers are male.       (Acceptability = − 1.29, Prevalence = 69.32)

Interestingly, the listed generics have a common attribute: They all generalize a particular feature of human beings. To account for such an exceptional pattern of those generics, we envisage the possibility that human-related generics behave differently from human-unrelated generics. Specifically, it is presumed that people are more inclined to reject the human-related generics than other types of generics for two possible reasons. First, people might have accumulated more sophisticated and rich knowledge about human beings in their daily lives, and therefore it is easy to recollect an example that counters what the generics in question describe, which eventually leads to rejecting the generalization. Second, people tend to be reluctant to the generalization that creates any kind of bias or stereotype about human beings, at least explicitly (Gilbert, 1951). Since further examination about the difference between human-related generics and other kinds of generics is not only beyond the scope of the present study but also inappropriate for the study due to the limited number of human-related generics, we leave more detailed exploration about the uniqueness of human-related generic to future studies.

**Table 6:** The metrics for the generics whose cue validity is not high

Target generics	Acceptability	Prevalence	Cue validity
Horses have four legs	2.86	96.86	.04
Needles are sharp	2.78	86.22	.58
Ducks lay eggs	2.76	70.00	.54
Fire trucks are red	2.61	83.65	.88
Snakes lay eggs	2.56	69.00	.83
Dogs have tails	2.55	89.57	1.04
US presidents are over 35	2.38	95.45	− 1.64
Preschoolers cannot vote	2.36	88.52	.42
Diapers are absorbent	2.36	92.45	1.08
Trumpets are loud	2.31	89.36	1.18
Goats have horns	2.29	75.74	.79
Sheep produce milk	2.26	54.24	.00
Feathers are light	2.14	93.26	.75
Moose have antlers	2.04	78.00	.96
Strokes kill people	2.00	63.52	− .41
Elk have antlers	1.93	72.91	1.23
Pigs suckle their young	1.71	63.96	1.13
Lead toys poison children	1.70	63.88	.63
Eggshells are white	1.63	67.04	.17
Rocking chairs are wooden	1.39	70.76	.52
Diapers are white	1.24	79.96	− .30
Plastic bags suffocate small children	1.14	52.30	1.13
Lions are male	.23	68.17	.33
Cats are white	− .27	38.17	.50
Ducks are female	− .30	52.45	− .91
Americans are brunettes	− .37	45.13	.25
Cars are yellow	− .43	23.43	.54
Teachers are female	− .50	62.79	.38
Humans are over 3 years old	− .67	61.71	.75
Tables are 10 feet long	− .77	29.29	1.17
Canadians are right-handed	− .87	72.22	− .29
Tigers are albino	− 1.17	21.57	− 1.30
Engineers are male	− 1.29	69.32	.38
Rooms are round	-1.41	28.17	− .67

### Summary

The results of the present study show that there exist flexible but systematic patterns of the way the acceptability of generics is influenced by two comparative probabilities. First of all, the acceptability of *IS_A* relational generics is mostly determined by prevalence, which has a strong negative correlation with cue validity. Second, if feature-describing generics contain high cue validity, then the acceptability is rated high, being mediated by prevalence. Third, the acceptability of feature-describing generics whose cue validity is not high is mostly determined by prevalence.

## General Discussion

Knowing the conditions that render sentences true is the central task of the formalization of their semantics. However, grasping the truth condition of generics seems intensely challenging primarily due to their vagueness and context-sensitivity. In an attempt to formalize the semantics of generics where such properties are taken into account, we proposed to account for the semantics of generics with flexible acceptance conditions that are defined in terms of two comparative probabilities—cue validity and prevalence. Within this approach, the semantic values of generics are to be represented on a continuous scale labeled as *acceptability* and the probability-based conditions are to be defined in a flexible but systematic way, enabling to accommodate diverse contexts.

In order to examine how such conditions should be constructed, we carried out a psycholinguistic experiment where three metrics—acceptability of generics, cue validity, and prevalence—are measured and closely investigated how the acceptability is influenced by the two types of comparative probabilities in different contexts. The findings from the experiment clearly revealed distinct contexts where two comparative probabilities put a different kind of influence on the acceptability of generic, indicating the existence of flexible but systematic patterns of the way in which probabilistic metrics influence the acceptability of generics.

First of all, the way in which comparative probabilities affect acceptability differs by the type of generics. Specifically, while the acceptability of *IS_A* relational generics is mostly accounted for by prevalence of no interactive relation with cue validity, the acceptability of feature-describing generics is explained by the interactive influence from the two types of comparative probabilities.

Motivated by the interaction effects observed between the cue validity and prevalence in explaining the acceptability of feature-describing generics, separate analyses on two types of feature-describing generics classified by the degree of cue validity were conducted to reveal two findings: (1) high cue validity endorses feature-describing generics acceptable despite the degree of the acceptability being marginally mediated by prevalence and (2) the acceptability of feature-describing generics whose cue validity is not high is mostly determined by prevalence, irrespective of cue validity.

With these findings taken together, we draw a conclusion that the acceptance condition of generics may be defined simply as follows.

*Condition 1* When a generics expresses an *IS_A* relation, then the acceptability is determined by the prevalence, without interactive relation with cue validity.

*Condition 2* When a generic describes a feature of certain category and it is with high cue validity, then the acceptability of the generic is endorsed despite being mediated by prevalence.

*Condition 3* When a generic describes a feature of a certain category and it is with low cue validity, then the acceptability of the generic is determined by prevalence.

In summary, the present study showed that comparative probabilities are by no means randomly related to the acceptability of generics. Rather, it seems that there are flexible but systematic patterns of the way in which the acceptability of generics relates to comparative probabilities, which may serve as a foundation for the formalized semantics of generics. Before concluding, we need to point out that our proposed model to measure the acceptability of generics may still be vulnerable to some counterexamples. However, we see that there is good potential and importance in our approach since it corroborates the possibility to find systematic patterns of the varying acceptance conditions of generics in terms of comparative probabilities. By extending the scope of the discussion and taking into account more diverse types of generics in future studies, a more solid formalization of the flexible semantics of generics could be established in terms of comparative probabilities.

## Footnotes

1 Generics can be definite, indefinite or bare plural sentences (Leslie & Lerner, 2016). In this paper, we only consider bare plural forms of generics.

2 Exploiting the gradable acceptability in place of discrete truth values to investigate the semantics of generics is not itself novel. Previous studies (Cohen, 2009; Prasada et al., 2013) have examined the semantics of generics in terms of gradual acceptability in place of discrete truth values that are traditionally used in formal semantics.

3 Prasada et al. (2013) have also classified generics into seven types. We also tested how the influence of comparative probabilities varies depending on the Prasada et al. (2013)’s classification, but no significant variation by the type was observed.
